# Cholecystectomy versus central obesity or insulin resistance in relation to the risk of nonalcoholic fatty liver disease: the third US National Health and Nutrition Examination Survey

**DOI:** 10.1186/s12902-019-0423-y

**Published:** 2019-09-02

**Authors:** Wenzhu Yue, Xingxing Sun, Tingting Du

**Affiliations:** 1 Department of Endocrinology, Tongji Hospital, Tongji Medical College, Huazhong University of Science and Technology, Wuhan, Hubei Province 430030 People’s Republic of China; 2 Department of Anesthesiology, Tongji Hospital, Tongji Medical College, Huazhong University of Science and Technology, Wuhan, 430030 China

**Keywords:** Nonalcoholic fatty liver disease, Cholecystectomy, Central obesity, Insulin resistance

## Abstract

**Background:**

Cholecystectomy, central obesity, and insulin resistance (IR) are established risk factors for nonalcoholic fatty liver disease (NAFLD). We aimed to examine the relative contributions and combined association of cholecystectomy and central obesity/IR with NAFLD risk.

**Methods:**

We conducted a cross-sectional analysis of data from the third National Health and Nutrition Examination Survey (NHANES III), in which ultrasonography was performed. Odds ratios (ORs) and 95% confidence intervals for NAFLD were estimated using logistic regression.

**Results:**

Cholecystectomy associated with a higher prevalence of NAFLD compared with gallstones among both centrally obese and non-centrally-obese subjects. Gallstones associated with a higher prevalence of NAFLD only in the presence of central obesity. In centrally obese participants, the OR increased from 2.67 (2.15–3.32) for participants without gallstone disease to 6.73 (4.40–10.29) for participants with cholecystectomy. In participants with cholecystectomy, the OR increased from 2.57 (1.35–4.89) for participants without central obesity to 6.73 (4.40–10.29) for centrally obese counterparts. We observed a modest increase in the risk of NAFLD with cholecystectomy compared with a large increase in the risk with IR or metabolic syndrome.

**Conclusion:**

The magnitude of the NAFLD risk contributed by cholecystectomy was similar to central obesity in combined analyses. The magnitude of the association with IR or metabolic syndrome was greater than with cholecystectomy.

**Electronic supplementary material:**

The online version of this article (10.1186/s12902-019-0423-y) contains supplementary material, which is available to authorized users.

## Background

Nonalcoholic fatty liver disease (NAFLD) forecasts an increased risk of cardiovascular disease (CVD) as well as premature mortality [1, 2]. Along with the burgeoning epidemics of obesity and metabolic syndrome, the worldwide prevalence of NAFLD is increasing rapidly, affecting between 15 and 40% of adults [3–5]. Recently, emerging evidence demonstrates that cholecystectomy is an independent risk factor for NAFLD [6], indicating cholecystectomy as a novel risk factor for NAFLD. Cholecystectomy and NAFLD share a similar etiology such as central obesity and insulin resistance (IR) [7]. Data on the interrelationship between central adiposity or IR and cholecystectomy on the NAFLD risk is limited. Considering that central adiposity, IR, and cholecystectomy are established independent risk factors for NAFLD, more robust investigation is necessary to determine the comparative importance and joint relationship of these factors on NAFLD. Therefore, we aimed to investigate the combined relationship of central obesity or IR and cholecystectomy with NAFLD to understand whether there is a difference in NAFLD burden from cholecystectomy according to obesity and IR status, using data from the Third National Health and Nutrition Examination Survey (NHANES III), which is a nationwide cross-sectional study with a nationally representative sample of US population.

## Methods

### Study population

We used data from the NHANES III, a cross-sectional health examination survey conducted by the National Center for Health Statistics of the Centers for Disease Control and Prevention in the United States between the years 1988 and 1994. Full details of the survey have been described elsewhere [8]. Briefly, the survey followed a complex stratified, multistage probability cluster sampling design to ensure that the sample is nationally representative of the civilian, noninstitutionalized US population. Participants were interviewed at home for basic sociodemographic and health-related information. After the in-home interview, participants are invited to attend a mobile examination center, where they underwent a set of standardized physical examinations and laboratory measurements. All data were collected according to the standardized NHANES protocols. Each adult participant provided a written informed consent and the survey was approved by the National Center for Health Statistics ethics review board.

Among the 14,745 participants who attended an examination at a mobile examination center, we restricted our analyses to non-pregnant adults who completed the physical and gallbladder ultrasound examination. We excluded 4671 participants from this study, comprising 3341 participants with alcohol consumption in amounts > 3 drinks/day for men (2212) or > 2 drinks/day for women (1219), 71 participants with serum hepatitis B surface antigen positivity, 390 participants with hepatitis C antibody positivity, 464 participants with iron overload (serum transferrin saturation > 50%), 814 participants with gallbladder lumen could not be adequately visualized on ultrasound, and 127 participants with ultrasound ungradable. As some individuals met more than one exclusion criteria, the remaining available 10,074 participants (4177 men and 5887 women) were included in our data analysis.

### Anthropometric and biochemical measurements

Body mass index (BMI) was calculated as weight (in kilograms) divided by the square of height (in meters). Waist circumference (WC) was measured with a steel measuring tape just above the iliac crest to the nearest 1 mm. Blood pressure (BP) was measured using mercury sphygmomanometers. The last two readings were averaged.

### Biochemical measurements

Participants were randomly assigned to morning (fast for at least 8 h prior to the examination), or afternoon/evening sessions (fast for 6 h before the examination). Persons without previously diagnosed diabetes were assigned to a morning session. Total cholesterol (TC), and HDL-cholesterol measurements were available for each examined person, regardless of fasting state, whereas triglycerides and insulin measurements were available only for persons examined in the morning session. Plasma glucose was measured by the modified hexokinase enzymatic assay (Cobas Mira Chemistry System; Roche Diagnostic Systems, Montclair, NJ). TC and triglycerides were measured enzymatically. HDL-cholesterol was measured by the heparin manganese precipitation method. Serum insulin was measured by radioimmunoassay using a doubleantibody batch method (Pharmacia Insulin RIA kit; Pharmacia Diagnostics, Uppsala, Sweden).

### Assessment of NAFLD

Ultrasound tests (Toshiba SSA-90A, Tustin, CA) were used for assessment of hepatic steatosis. Archived videotapes on gallbladder ultrasounds were reviewed between 2009 and 2010 to ascertain the presence of fat within the hepatic parenchyma. The diagnosis of fatty liver was based on the following five criteria [9]: the brightness of liver parenchymal, presence of liver to kidney contrast, presence of echogenic walls within the small intrahepatic vessels, presence of deep beam attenuation, and definition of gallbladder walls. NAFLD was initially categorized as a 4-level classification (none, mild, moderate, or severe) and then recoded as a 2-level classification (none to mild or moderate to severe), which was the classification used for the current analysis. For the two-level hepatic steatosis categorization, the intrarater and interrater k statistics were 0.77 (95% confidence interval [CI] 0.73–0.82) and 0.70 (0.64–0.76), respectively.

### Assessment of gallstone disease

Gallstone disease was defined as ultrasound-documented gallstones (the presence of echoes within the gallbladder and acoustic shadowing) or evidence of a cholecystectomy (the presence of a right upper quadrant or epigastric scar and together with the absence of a gallbladder) using the standard criteria [10]. For the diagnosis of gallbladder disease, the ultrasonographer and reviewing radiologist k statistics were 0.97.

### Assessment of insulin resistance

Homeostasis model assessment of insulin resistance (HOMA-IR) was calculated by the formula: HOMA-IR = fasting insulin (micro-international units per milliliter) × fasting glucose (millimoles per liter)/22.5. Quartiles of the HOMA-IR were calculated in persons with fasting glucose < 7.0 mmol/l. IR was defined as having HOMA-IR in the upper quartile of the HOMA index.

### Definition of metabolic syndrome

The metabolic syndrome (MetS) was defined according to the consensus criteria in 2009 [11]. Persons with MetS are those with the presence of three or more of the following criteria: central obesity, WC ≥ 102 cm in men and ≥ 88 cm in women; Triglycerides ≥1.7 mmol/l or on lipid-lowering drug treatment in a patient with a history of dyslipidemia; HDL-cholesterol < 1.0 mmol/l in men and < 1.3 mmol/l in women; Systolic/diastolic BP ≥ 130/85 mmHg or use of antihypertensive drug treatment in a patient with a history of hypertension; or fasting glucose ≥5.6 mmol/l.

### Statistical analysis

Complex survey procedures in SAS 9.2 (SAS Institute, Inc., Cary, NC) were performed for all analyses. Sample weights were incorporated to produce nationally representative estimates. Since BMI is a poor indicator of body fat distribution [12], we adopt WC to define central obesity according to International Diabetes Federation criteria [11]. Based on cross-classification of the WC and gallstone disease status, participants were categorized into 6 mutually exclusive groups (no gallstone disease with or without central obesity, gallstones with or without central obesity, cholecystectomy with or without central obesity). Triglycerides, insulin and HOMA-IR were log-transformed (natural logarithm) to approximate normal distributions. Continuous variables were expressed as means and standard errors (SE). Categorical variables were expressed as percentages. ANOVA was applied to compare differences in means across groups. A Chi-square test was performed to assess differences of proportions across groups. Bonferroni correction was applied to adjust *P* values for multiple comparisons. Odds ratios (ORs) for NAFLD in each group were determined using logistic regression analysis, and the group without gallstone disease and central obesity was used as the reference. A logistic regression model was used to examine the joint associations of gallstone disease and central obesity status with NAFLD risk to determine whether the associations of gallstone disease with NAFLD differed based on the presence of central obesity, MetS, or IR. Significance was accepted at a two-tailed *P* < 0.05.

## Results

The clinical characteristics of the study population classified according to WC and gallstone disease status were shown in Table 1. Centrally obese subjects with cholecystectomy were older, more likely to be women, and had higher levels of BMI, WC, systolic/diastolic BP, plasma glucose, TC, triglycerides, insulin, and HOMA-IR, and lower levels of HDL-cholesterol than non-centrally-obese subjects without gallstone disease. Centrally obese subjects with cholecystectomy was older age, less educated, more likely to be women, had higher levels of BMI, insulin, and HOMA-IR compared with centrally obese subjects without gallstone disease. There was no difference in almost all the CVD risk factors between subjects with gallstones and subjects with cholecystectomy, irrespective of whether individuals were centrally obese. Among subjects with cholecystectomy, centrally obese subjects were less likely to be educated, had higher levels of BMI, WC, systolic/diastolic BP, plasma glucose, TC, triglycerides, insulin, and HOMA-IR compared with non-centrally-obese subjects.

**Table 1:** Age- and sex- standardized characteristics of participants by central obesity and gallstone disease status

	No central obesity			Central obesity		
	No gallstone disease (*N* = 4917)	Gallstones (*N* = 324)	Cholecystectomy (*N* = 184)	No gallstone disease (*N* = 3443)	Gallstones (*N* = 618)	Cholecystectomy (*N* = 588)
Women (%)	42.8 ± 1.5	51.5 ± 3.8	73.3 ± 5.0*^£^	59.3 ± 1.4*	69.3 ± 3.1*^$^	77.9 ± 2.8*^#^
Race ethnicity (%)						
Non-Hispanic white	86.6 ± 1.5	92.2 ± 2.4	89.7 ± 3.3	82.4 ± 1.6	84.9 ± 2.1	84.1 ± 2.6
Non-Hispanic black	6.1 ± 0.9	3.0 ± 1.2	4.9 ± 1.6	11.2 ± 1.0*	7.4 ± 1.1^$#^	4.0 ± 1.0^#&^
Mexican American	1.0 ± 0.3	1.7 ± 0.5	3.0 ± 1.0	2.6 ± 0.3*	4.3 ± 0.7*^$^	4.4 ± 0.7*
Smoking (%)	59.5 ± 2.9	64.4 ± 4.5	63.5 ± 4.7	60.4 ± 2.0	53.8 ± 3.1	55.9 ± 3.2
Age (years)	65.6 ± 0.2	66.9 ± 0.4*	66.8 ± 0.5	66.6 ± 0.2*	67.6 ± 0.3*	68.3 ± 0.3*^#^
Education (years)	12.0 ± 0.2	11.7 ± 0.3	11.6 ± 0.3	11.4 ± 0.2*	11.5 ± 0.2	10.6 ± 0.2*^#+&^
Body mass index (kg/m^2^)	22.3 ± 0.1	22.4 ± 0.2	23.0 ± 0.3	30.3 ± 0.2*	30.9 ± 0.4*^$^	32.3 ± 0.5*^#+^
Waist circumference (cm)	85.0 ± 0.3	85.2 ± 0.7	83.4 ± 0.9	105.5 ± 0.4*	106.4 ± 0.8*^$^	108.0 ± 1.0*^+^
SBP (mmHg)	132.6 ± 0.4	133.2 ± 1.2	130.4 ± 1.7	137.8 ± 0.5*	139.0 ± 1.0*^$^	140.1 ± 0.9*^+^
DBP (mmHg)	73.0 ± 0.3	71.5 ± 0.7	70.2 ± 1.0*	76.8 ± 0.3*	76.5 ± 0.6*^$^	75.4 ± 0.6*^+^
Plasma glucose (mmol/l)	5.5 ± 0.1	5.6 ± 0.1	5.5 ± 0.1	6.0 ± 0.1*	6.1 ± 0.1*	6.4 ± 0.2*^+^
Total cholesterol (mmol/l)	5.7 ± 0.0	5.5 ± 0.1	5.6 ± 0.1	6.0 ± 0.0*	5.9 ± 0.1*^$^	6.0 ± 0.1*^+^
^¥^Ln Triglycerides (mmol/l)	0.3 ± 0.0	0.3 ± 0.1	0.3 ± 0.1	0.6 ± 0.0*	0.6 ± 0.1*^$^	0.7 ± 0.1*^+^
LDL-cholesterol (mmol/l)	3.6 ± 0.1	3.4 ± 0.1	3.4 ± 0.1	3.9 ± 0.0*	3.7 ± 0.1	3.7 ± 0.1
HDL-cholesterol (mmol/l)	1.4 ± 0.0	1.4 ± 0.0	1.4 ± 0.1	1.3 ± 0.0*	1.3 ± 0.0*	1.3 ± 0.0*
^¥^Ln Insulin (pmol/l)	3.8 ± 0.0	3.8 ± 0.1	3.8 ± 0.1	4.3 ± 0.0*	4.4 ± 0.0*^$^	4.5 ± 0.1*^#+^
^¥^Ln HOMA-IR	0.6 ± 0.0	0.7 ± 0.1	0.6 ± 0.1	1.2 ± 0.1*	1.3 ± 0.1*^$^	1.4 ± 0.1*^#+^

Characteristics were age and sex standardized to the 2000 US population distribution. Mean age and % women were not standardized

Data are means±SEs or percentages. ^¥^Measured in non-diagnosed diabetics who were examined in the morning after an overnight fast

*SBP* systolic blood pressure, *DBP* diastolic blood pressure, *HOMA-IR* Homeostasis model assessment of insulin resistance

^*^P < 0.0056 compared with the group without central obesity and without gallstone disease; ^#^*P* < 0.0056 compared with the group with central obesity but without gallstone disease; ^$^*P* < 0.0056 compared with the group without central obesity but with gallstones; ^†^*P* < 0.0056 compared with the group without central obesity but with cholecystectomy; ^£^*P* < 0.0056 compared with the group without central obesity but with gallstones

^&^*P* < 0.0056 compared with the group with central obesity and with gallstones

**Fig. 1:**
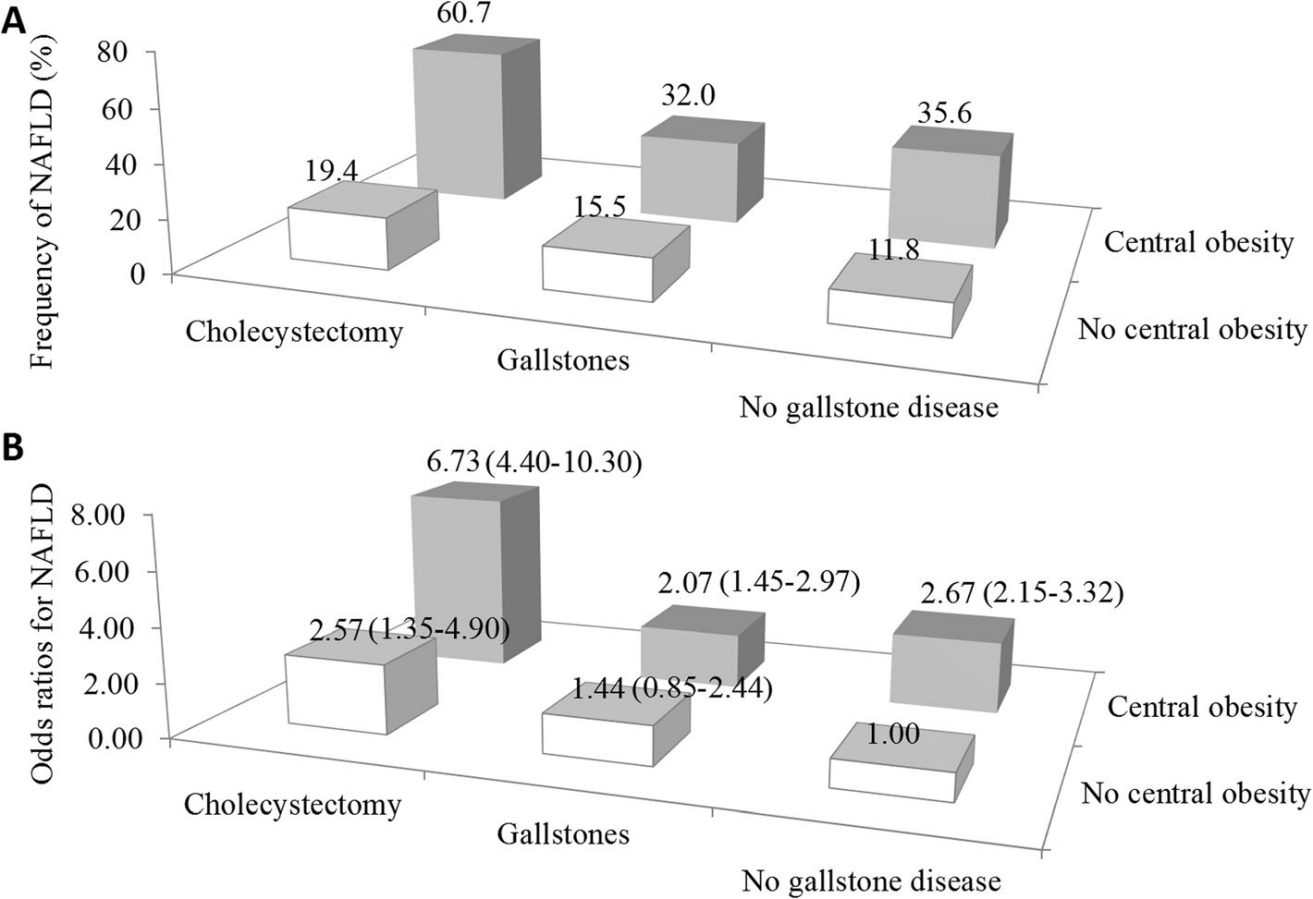
Combined effect of gallstone disease and central obesity on the risk of non-alcoholic fatty liver disease (NAFLD). Age-and sex-standardized prevalence of NAFLD by central obesity and gallstone disease status (**a**). Combined effect of gallstone disease and central obesity on the odds of NAFLD (**b**). Cholecystectomy associated with a higher prevalence of NAFLD compared with gallstones among both centrally obese and non-central-obese subjects. Gallstones associated with a higher prevalence of NAFLD only in the presence of central obesity. Odds ratios (95% confidence intervals) of NAFLD for participants categorized by cross-classification of central obesity and gallstone disease status were adjusted for age, sex, race ethnicity, smoking and drinking status, education level, systolic blood pressure, hemoglobin A1c, total cholesterol, and HDL-cholesterol

The prevalence of NAFLD showed an increasing trend from the group with neither central obesity nor cholecystectomy to the group with both central obesity and cholecystectomy (Fig. 1a). Cholecystectomy associated with a higher prevalence of NAFLD compared with gallstones among both centrally obese and non-centrally-obese subjects. Gallstones associated with a higher prevalence of NAFLD only in the presence of central obesity.

Centrally obese participants had an increased risk of NAFLD with a multivariate OR of 2.53 (2.02–3.17) (Table 2). Further adjustment for gallstone disease status did not change the association (Table 2). In multivariate-adjusted analyses, gallstones were not independently related to NAFLD (Table 3). Participants with a cholecystectomy had an increased risk of NAFLD with a multivariate OR of 2.61 (1.89–3.61). Further adjustment for WC did not appreciably change the strength of the association. There was no significant overall effect modification of cholecystectomy by WC when the interaction term WC*gallstone disease status was analyzed (*P* = 0.33).

**Table 2:** Adjusted odds ratios (with 95% confidence intervals) for the central obesity-related risk of nonalcoholic fatty liver disease

	No central obesity	Central obesity
Multivariate adjusted^a^	1	2.53 (2.02–3.17)
Multivariate adjusted^b^	1	2.53 (2.03–3.16)

^a^Adjusted for age, sex, race ethnicity, smoking and drinking status, education level, systolic blood pressure, hemoglobin A1c, total cholesterol, and HDL-cholesterol

^b^Adjusted for aforementioned covariates and gallstone disease status

**Table 3:** Adjusted odds ratios (with 95% confidence intervals) for the gallstones- and cholecystectomy-related the risk of nonalcoholic fatty liver disease

	No gallstone disease	Gallstones	Cholecystectomy
Multivariate adjusted^a^	1	0.95 (0.68–1.31)	2.61 (1.89–3.61)
Multivariate adjusted^b^	1	0.90 (0.66–1.22)	2.56 (1.85–3.53)

^a^Adjusted for age, sex, race ethnicity, smoking and drinking status, education level, systolic blood pressure, hemoglobin A1c, total cholesterol, and HDL-cholesterol

^b^Adjusted for aforementioned covariates and waist circumference

Participants with both central obesity and cholecystectomy had a greater risk of NAFLD than the sum of their individual risks (Fig. 1b). In the multivariate-adjusted model, cholecystectomy had an effect on the risk of NAFLD similar to central obesity. For example, in centrally obese participants, the OR increased from 2.67 (2.15–3.32) for participants without gallstone disease to 6.73 (4.40–10.29) for participants with cholecystectomy. In participants with cholecystectomy, the OR increased from 2.57 (1.35–4.89) for participants without central obesity to 6.73 (4.40–10.29) for centrally obese counterparts. We also found that cholecystectomy had an effect on the risk of NAFLD similar to central obesity assessed by WC/hip circumference (HC). For example, participants with only WC/HC defined centrally obese participants were at 1.94 -fold risk for NAFLD, and participants with only cholecystectomy were at 2.31-fold risk of NAFLD (Additional file 1: Figure S1).

**Fig. 2:**
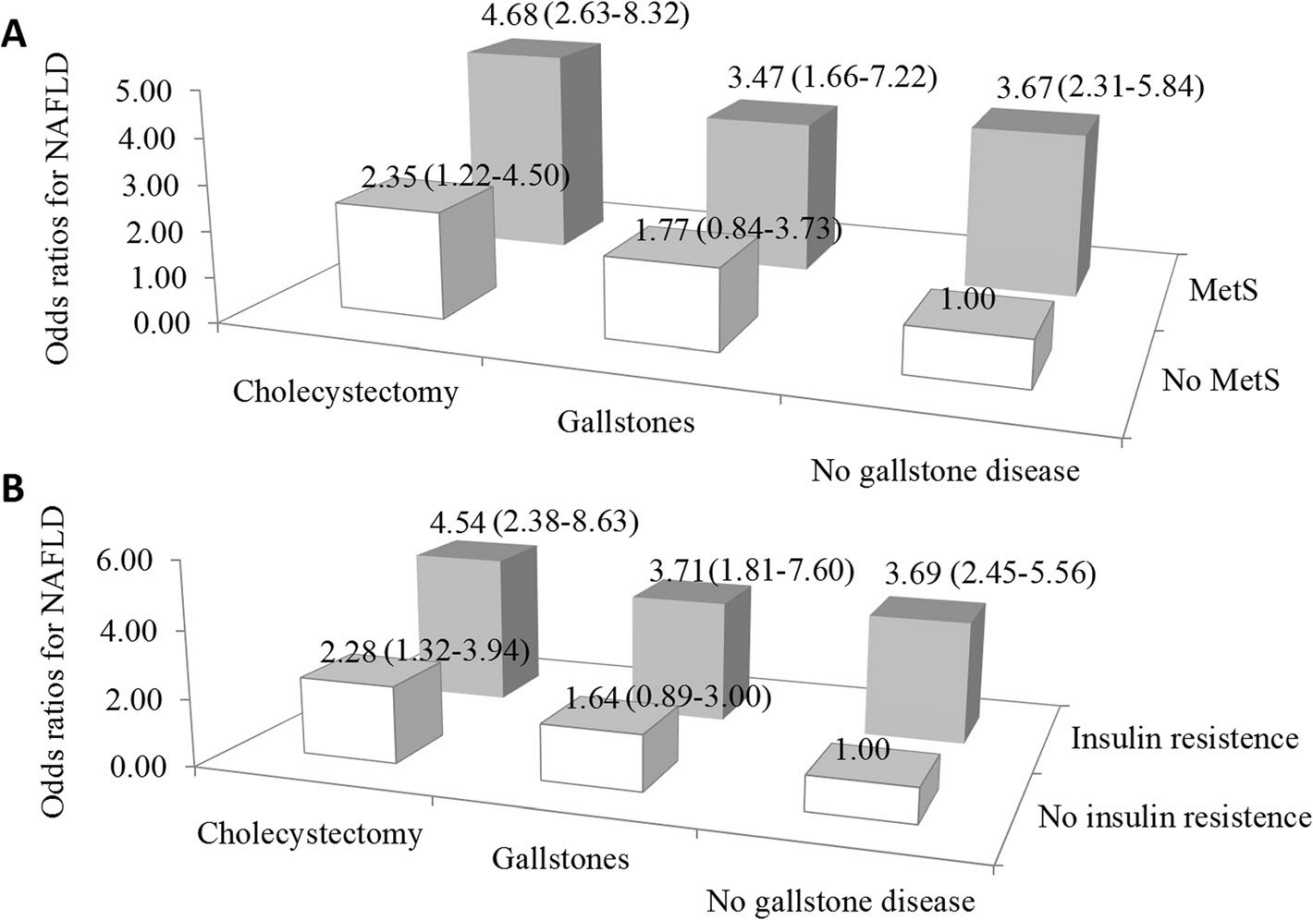
Combined effect of gallstone disease and metabolic syndrome (MetS) or combined effect of gallstone disease and insulin resistance on the risk of non-alcoholic fatty liver disease (NAFLD). Combined effect of gallstone disease and MetS on the odds of NAFLD (**a**). Combined effect of gallstone disease and insulin resistance on the odds of NAFLD (**b**). Odds ratios (95% confidence intervals) of NAFLD were adjusted for age, sex, race ethnicity, smoking and drinking status, education level, hemoglobin A1c, and total cholesterol

Since triglycerides and insulin measurements were available only for persons without known diabetes who examined in the morning session. A secondary analysis was conducted among 5521 participants who fasted for 9–24 h. Participants were then categorized according to IR and gallstone disease status and again according to MetS and gallstone disease status. Cholecystectomy and IR or MetS had synergetic effects on the risk of NAFLD. Both IR and MetS tended to have a greater influence on NAFLD risk than cholecystectomy in these analyses (Fig. 2).

**Fig. 3:**
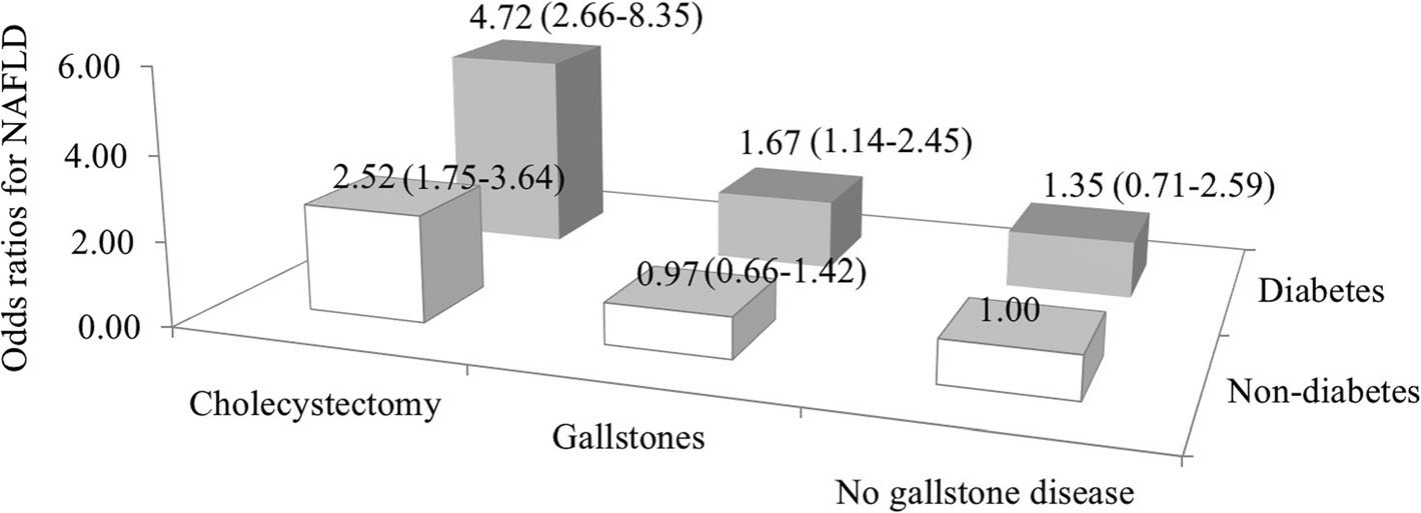
Combined effect of gallstone disease and diabetes on the risk of non-alcoholic fatty liver disease (NAFLD). Odds ratios (95% confidence intervals) of NAFLD were adjusted for age, sex, race ethnicity, smoking and drinking status, education level, total cholesterol, HOMA-IR, waist circumference, hemoglobin A1c, diabetes duration, and anti-diabetic drugs

After adjusting for age, sex, race ethnicity, smoking and drinking status, education level, total cholesterol, HOMA-IR, waist circumference, hemoglobin A1c, diabetes duration, and anti-diabetic drugs, cholecystectomy associated with a higher prevalence of NAFLD compared with gallstones among both diabetic and non-diabetic subjects. Gallstones associated with a higher prevalence of NAFLD only in the presence diabetes (Fig. 3).

## Discussion

Consistent with previous studies, we found that WC, IR, MetS, and cholecystectomy independently contributed to the risk of NAFLD [6, 13]. This study further revealed that the magnitude of the risk contributed by central obesity was similar to that by cholecystectomy. We also observed that the magnitude of the association with IR or MetS was greater than with cholecystectomy in combined analyses. This study also revealed gallstones were a risk factor for NAFLD only in the presence of central obesity, IR or MetS. In this study, comparative and joint analyses broaden our understanding of risk factors’ relative influence on NAFLD.

In the present study, those with gallstones but without central obesity, IR or MetS did not have increased risk of NAFLD. However, cholecystectomy was associated with increased risk of NAFLD, regardless of central obesity, IR or MetS status, supporting the notion that cholecystectomy may itself represent a risk factor for NAFLD [6]. Our findings suggested that the effects of gallstones on NAFLD were mediated primarily by the presence of central obesity, IR or MetS. These results are consistent with previous reports in which gallstones were unrelated to NAFLD after correction for central obesity or metabolic risk factors [6, 14]. It is not unexpected that different relationships were observed between NAFLD and gallstones and cholecystectomy, as the latter measures different aspects of hepatic lipid metabolism and biliary lipid secretion, and represents the loss of the metabolic functions in the absence of a gallbladder [15, 16]. Ablation of the gallbladder eliminates the formation of cholesterol solid-plate crystals, which form in the gallbladder and is not able to be absorbed by the intestine [17]. Therefore, cholecystectomy can increase the proportion of cholesterol that is reabsorbed and delivered to the liver, facilitating an increase in hepatic triglycerides concentration. The gallbladder is critical to regulating the daily cycling of bile acids (BAs) within the enterohepatic circulation. The rhythm and intensity of BAs secretion from a healthy functional gallbladder are in synchrony with food intake. Cholecystectomy may alter the rhythm of BAs flux and its receptor activation, although the basal BAs output is higher than normal, the release of BAs is not enough to balance the sharp fluctuation of lipid and glucose levels after food intake [18]. Following the removal of the gallbladder, BAs are continuously secreted into the small intestine, and the BAs pool circulates faster, inducing increased cycling of the BA pool and thus expose the liver to a greater flux of BAs and the occurrence of metabolic derangements. Emerging evidence suggested that in addition to their well-established roles in absorption of dietary lipids, BAs are also signaling molecules that act as ligands of the nuclear receptor farnesoid X receptor and the G-protein-coupled BA receptor TGR5, which modulate complex enterohepatic and systemic lipid and glucose metabolism [19, 20]. Moreover, ablation of the gallbladder could remove the protective metabolic activity of the gallbladder mucosa, which can secret fibroblast growth factor 19 that has a role in the negative feedback regulation of BA synthesis and can inhibit hepatic fatty acid synthesis [21, 22]. The loss of this metabolic activity and changes in following cholecystectomy renders patients prone to an increased NAFLD risk.

One study using the same data have reported that the odds of NAFLD did not differ based on time since cholecystectomy [6]. There are two longitudinal studies assessed the risk of liver steatosis after cholecystectomy [23, 24]. One study reported that hepatic steatosis developed 3 months after cholecystectomy [23]. Another study noted that hepatic fat content was significantly increased 24 months after cholecystectomy [24]. Hence, changes in lipid metabolism and bile acids secretion after cholecystectomy can affect the structure of the liver. However, the exact time it takes to develop NAFLD remains to be determined.

Data on the relative influence of central obesity and cholecystectomy on NAFLD risk are sparse. The present study indicated that the increased NAFLD risk was similar between non-central-obese individuals with cholecystectomy and centrally obese individuals without cholecystectomy, suggesting that the clinical evaluation of cholecystectomy play similar important role to the diagnosis of central obesity in NAFLD risk stratification. Our data also showed additive effects of cholecystectomy above central obesity for NAFLD risk. Although there are many theories, the mechanism by which central obesity affects cholecystectomy remains poorly understood. One possible explanation may be that WC and cholecystectomy are 2 independent variables, they interact with each other and contribute to some different pathogenesis leading to NAFLD. This is suggested by the no attenuation of the effects of cholecystectomy on NAFLD risk by adjusting for WC in our analyses. Since adipose tissue lipolysis supplies the majority of free fatty acids that subsequently are esterified to form hepatic triglycerides [25], obesity has been well recognized for its strong association with fat accumulation in the liver [26]. Increased cycling of the BA pool after cholecystectomy could favor even a greater increase of body cholesterol synthesis and hypersecretion of biliary cholesterol observed in obesity, increasing the exposure of the liver to a higher flux of triglycerides and thus exacerbating hepatic steatosis. Therefore, cholecystectomy can exaggerate the adverse effects of central obesity on NAFLD risk.

Since symptoms are believed to be uncommon and transitory after removal of gallbladder, cholecystectomy has become one of the most frequently performed surgical procedures for treatment for gallstone disease. We observed that the magnitude of the association with NAFLD risk was similar for WC and cholecystectomy. Hence, cholecystectomy may not be innocuous.

The present study indicated that cholecystectomy had relatively small effects on NAFLD risk than IR or MetS. Alterations in gut microbiota have been linked to host insulin resistance, and diabetes [27, 28]. Emerging evidence reported that altered composition of gut microbiota was noted in patients who underwent cholecystectomy; the community diversity of intestinal microbiota in cholecystectomized patients was decreased and Bacteroidetes were increased compared with healthy population [29, 30]. A human study found that low richness of gut microbiome plays a role in insulin resistance [28]. Taken together, alterations in gut microbiota after cholecystectomy may contribute to the involvement of insulin resistance and metabolic abnormalities. Moreover, due to the cross-sectional nature of the data, any temporal effect of cholecystectomy versus IR or MetS on risk of NAFLD could not be determined.

According to guideline, people with asymptomatic gallbladder stones found in a normal gallbladder and normal biliary tree do not need treatment unless they develop symptoms. Symptomatic gallbladder stones whether symptomatic or asymptomatic should be offered cholecystectomy [31]. Hence, for patients with gallstones together with other risk factors such as obesity, hyperlipidemia, insulin resistance, and T2DM, which are predisposed to confer an increased NAFLD risk, comprehensive treatment including lifestyle interventions, BP, and glucose control should be take into consideration to keep body weight, BP and glucose in optimal levels and thus to avoid the occurrence of a symptomatic gallbladder.

Limitations of our study include the use of ultrasound to detect NAFLD. Although ultrasonography is a reasonably accurate technique for detecting modest amounts of liver fat (> 30% liver fat infiltration), participants with minor amounts of fatty infiltration might not have been captured. Furthermore, lack of information on residual confounding variables such as body fat percent prevented us from being able to assess these variables as potential confounders; however, major confounding factors have been controlled for in our multivariate models. These limitations are balanced by the benefits of using a nationally representative sample of US adults, particularly the ability to generalize the results to a national population. On the other hand, this study is the first human study to compare the importance of cholecystectomy and obesity on NAFLD risk.

## Conclusions

The present study demonstrates that central obesity, IR, MetS, and cholecystectomy play important roles in NAFLD. The magnitude of the association of NAFLD with cholecystectomy was similar to that with central obesity. The magnitude of the association with NAFLD risk was much greater for IR or MetS than for cholecystectomy. By understanding of the relative and combined influence of cholecystectomy and central obesity/IR on NAFLD, we may improve our ability to risk stratify patients and in turn may reduce the NAFLD incidence.

## Additional file

Additional file 1: **Figure S1.** Combined effect of gallstone disease and central obesity defined by waist circumference/hip circumference on the risk of non-alcoholic fatty liver disease (NAFLD). Cholecystectomy associated with a higher prevalence of NAFLD compared with gallstones among both centrally obese and non-central-obese subjects. Gallstones associated with a higher prevalence of NAFLD only in the presence of central obesity. Odds ratios (95% confidence intervals) of NAFLD for participants categorized by cross-classification of central obesity and gallstone disease status were adjusted for age, sex, race ethnicity, smoking and drinking status, education level, systolic blood pressure, hemoglobin A1c, total cholesterol, and HDL-cholesterol. (TIF 2903 kb)

## Data Availability

The datasets analyzed during the current study are available on the website https://www.cdc.gov/nchs/nhanes/about_nhanes.htm#data.

## Abbreviations

BAs: Bile acids
BMI: Body mass index
BP: Blood pressure
CVD: Cardiovascular disease
HOMA-IR: Homeostasis model assessment of insulin resistance
IR: Insulin resistance
MetS: Metabolic syndrome
NAFLD: Nonalcoholic fatty liver disease
OR: Odds ratio
SE: Standard errors
TC: Total cholesterol
WC: Waist circumference

## References

[1] Stepanova M, Younossi ZM (2012). Independent association between nonalcoholic fatty liver disease and cardiovascular disease in the US population. Clin Gastroenterol Hepatol.

[2] Ong JP, Pitts A, Younossi ZM (2008). Increased overall mortality and liver-related mortality in non-alcoholic fatty liver disease. J Hepatol.

[3] Browning JD, Szczepaniak LS, Dobbins R, Nuremberg P, Horton JD, Cohen JC (2004). Prevalence of hepatic steatosis in an urban population in the United States: impact of ethnicity. Hepatology..

[4] Chalasani N, Younossi Z, Lavine JE, Diehl AM, Brunt EM, Cusi K (2012). The diagnosis and management of non-alcoholic fatty liver disease: practice guideline by the American Gastroenterological Association, American Association for the Study of Liver Diseases, and American College of Gastroenterology. Gastroenterology..

[5] Vernon G, Baranova A, Younossi ZM (2011). Systematic review: the epidemiology and natural history of non-alcoholic fatty liver disease and non-alcoholic steatohepatitis in adults. Aliment Pharmacol Ther.

[6] Ruhl CE, Everhart JE (2013). Relationship of non-alcoholic fatty liver disease with cholecystectomy in the US population. Am J Gastroenterol.

[7] Nervi F, Miquel JF, Alvarez M, Ferreccio C, Garcia-Zattera MJ, Gonzalez R (2006). Gallbladder disease is associated with insulin resistance in a high risk Hispanic population. J Hepatol.

[8] Plan and operation of the Third National Health and Nutrition Examination Survey, 1988–94. Series 1: programs and collection procedures. Vital Health Stat 1. 1994;(32):1–407. 7975354

[9] Lazo M, Hernaez R, Bonekamp S, Kamel IR, Brancati FL, Guallar E (2011). Non-alcoholic fatty liver disease and mortality among US adults: prospective cohort study. BMJ (Clinical research ed).

[10] Everhart JE, Khare M, Hill M, Maurer KR (1999). Prevalence and ethnic differences in gallbladder disease in the United States. Gastroenterology..

[11] Alberti KG, Eckel RH, Grundy SM, Zimmet PZ, Cleeman JI, Donato KA (2009). Harmonizing the metabolic syndrome: a joint interim statement of the international diabetes federation task force on epidemiology and prevention; National Heart, Lung, and Blood Institute; American Heart Association; world heart federation; international atherosclerosis society; and International Association for the Study of obesity. Circulation..

[12] Rankinen T, Kim SY, Perusse L, Despres JP, Bouchard C (1999). The prediction of abdominal visceral fat level from body composition and anthropometry: ROC analysis. Int J Obes Relat Metab Disord.

[13] Speliotes EK, Massaro JM, Hoffmann U, Vasan RS, Meigs JB, Sahani DV (2010). Fatty liver is associated with dyslipidemia and dysglycemia independent of visceral fat: the Framingham heart study. Hepatology..

[14] Ruhl CE, Everhart JE (2011). Gallstone disease is associated with increased mortality in the United States. Gastroenterology..

[15] Kullak-Ublick GA, Paumgartner G, Berr F (1995). Long-term effects of cholecystectomy on bile acid metabolism. Hepatology..

[16] Roda E, Aldini R, Mazzella G, Roda A, Sama C, Festi D (1978). Enterohepatic circulation of bile acids after cholecystectomy. Gut..

[17] Wang DQ, Lee SP (2008). Physical chemistry of intestinal absorption of biliary cholesterol in mice. Hepatology..

[18] Chen Y, Wu S, Tian Y (2018). Cholecystectomy as a risk factor of metabolic syndrome: from epidemiologic clues to biochemical mechanisms. Lab Invest.

[19] Trauner M, Claudel T, Fickert P, Moustafa T, Wagner M (2010). Bile acids as regulators of hepatic lipid and glucose metabolism. Digestive diseases (Basel, Switzerland).

[20] Matsubara T, Li F, Gonzalez FJ (2013). FXR signaling in the enterohepatic system. Mol Cell Endocrinol.

[21] Potthoff MJ, Kliewer SA, Mangelsdorf DJ (2012). Endocrine fibroblast growth factors 15/19 and 21: from feast to famine. Genes Dev.

[22] Zweers SJ, Booij KA, Komuta M, Roskams T, Gouma DJ, Jansen PL (2012). The human gallbladder secretes fibroblast growth factor 19 into bile: towards defining the role of fibroblast growth factor 19 in the enterobiliary tract. Hepatology..

[23] Yun S, Choi D, Lee KG, Kim HJ, Kang BK, Kim H (2016). Cholecystectomy causes ultrasound evidence of increased hepatic steatosis. World J Surg.

[24] Cortes V, Quezada N, Uribe S, Arrese M, Nervi F (2017). Effect of cholecystectomy on hepatic fat accumulation and insulin resistance in non-obese Hispanic patients: a pilot study. Lipids Health Dis.

[25] Donnelly KL, Smith CI, Schwarzenberg SJ, Jessurun J, Boldt MD, Parks EJ (2005). Sources of fatty acids stored in liver and secreted via lipoproteins in patients with nonalcoholic fatty liver disease. J Clin Invest.

[26] Marchesini G, Brizi M, Bianchi G, Tomassetti S, Bugianesi E, Lenzi M (2001). Nonalcoholic fatty liver disease: a feature of the metabolic syndrome. Diabetes..

[27] Le Chatelier E, Nielsen T, Qin J, Prifti E, Hildebrand F, Falony G (2013). Richness of human gut microbiome correlates with metabolic markers. Nature..

[28] Pedersen HK, Gudmundsdottir V, Nielsen HB, Hyotylainen T, Nielsen T, Jensen BA (2016). Human gut microbes impact host serum metabolome and insulin sensitivity. Nature..

[29] Keren N, Konikoff FM, Paitan Y, Gabay G, Reshef L, Naftali T (2015). Interactions between the intestinal microbiota and bile acids in gallstones patients. Environ Microbiol Rep.

[30] Wang W, Wang J, Li J, Yan P, Jin Y, Zhang R (2018). Cholecystectomy damages aging-associated intestinal microbiota construction. Front Microbiol.

[31] Warttig S, Ward S, Rogers G (2014). Diagnosis and management of gallstone disease: summary of NICE guidance. BMJ (Clinical research ed).

