# Supplementary figures and images for: Cholecystectomy versus central obesity or insulin resistance in relation to the risk of nonalcoholic fatty liver disease: the third US National Health and Nutrition Examination Survey
Source: BMC Endocr Disord. 2019 Sep 2;19:95. doi: 10.1186/s12902-019-0423-y (PMC6720375; doi:10.1186/s12902-019-0423-y)

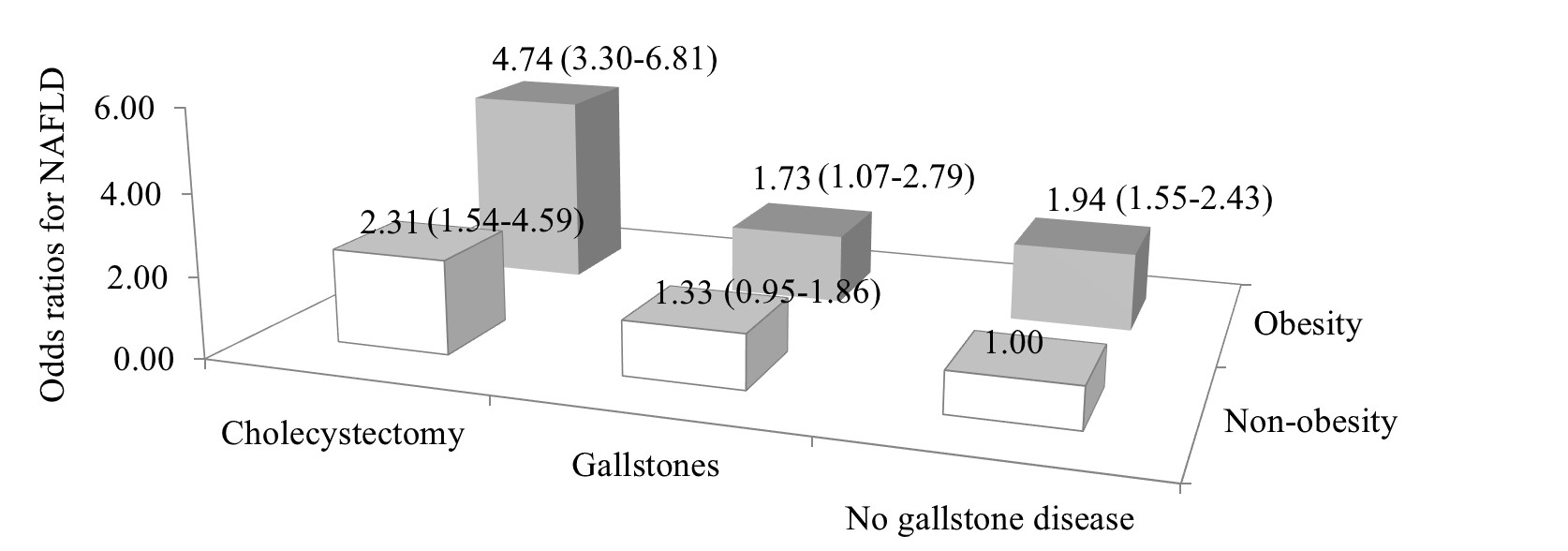

Supplement: Supplementary file 1 — Figure S1. Combined effect of gallstone disease and central obesity defined by waist circumference/hip circumference on the risk of non-alcoholic fatty liver disease (NAFLD). Cholecystectomy associated with a higher prevalence of NAFLD compared with gallstones among both centrally obese and non-central-obese subjects. Gallstones associated with a higher prevalence of NAFLD only in the presence of central obesity. Odds ratios (95% confidence intervals) of NAFLD for participants categorized by cross-classification of central obesity and gallstone disease status were adjusted for age, sex, race ethnicity, smoking and drinking status, education level, systolic blood pressure, hemoglobin A1c, total cholesterol, and HDL-cholesterol. (TIF 2903 kb) [file 12902_2019_423_MOESM1_ESM.tif]
